# Regulation of Ste20-like kinase, SLK, activity: Dimerization and activation segment phosphorylation

**DOI:** 10.1371/journal.pone.0177226

**Published:** 2017-05-05

**Authors:** Andrey V. Cybulsky, Julie Guillemette, Joan Papillon, Nihad T. Abouelazm

**Affiliations:** 1 Department of Medicine, McGill University Health Centre Research Institute, McGill University, Montreal, Quebec, Canada; 2 Department of Clinical Pharmacology, Faculty of Medicine, Alexandria University, Alexandria, Egypt; Institut de Genetique et Developpement de Rennes, FRANCE

## Abstract

The Ste20-like kinase, SLK, has diverse cellular functions. SLK mediates organ development, cell cycle progression, cytoskeletal remodeling, cytokinesis, and cell survival. Expression and activity of SLK are enhanced in renal ischemia-reperfusion injury, and overexpression of SLK was shown to induce apoptosis in cultured glomerular epithelial cells (GECs) and renal tubular cells, as well as GEC/podocyte injury in vivo. The SLK protein consists of a N-terminal catalytic domain and an extensive C-terminal domain, which contains coiled-coils. The present study addresses the regulation of SLK activity. Controlled dimerization of the SLK catalytic domain enhanced autophosphorylation of SLK at T183 and S189, which are located in the activation segment. The full-length ectopically- and endogenously-expressed SLK was also autophosphorylated at T183 and S189. Using ezrin as a model SLK substrate (to address exogenous kinase activity), we demonstrate that dimerized SLK 1–373 or full-length SLK can effectively induce activation-specific phosphorylation of ezrin. Mutations in SLK, including T183A, S189A or T193A reduced T183 or S189 autophosphorylation, and showed a greater reduction in ezrin phosphorylation. Mutations in the coiled-coil region of full-length SLK that impair dimerization, in particular I848G, significantly reduced ezrin phosphorylation and tended to reduce autophosphorylation of SLK at T183. In experimental membranous nephropathy in rats, proteinuria and GEC/podocyte injury were associated with increased glomerular SLK activity and ezrin phosphorylation. In conclusion, dimerization via coiled-coils and phosphorylation of T183, S189 and T193 play key roles in the activation and signaling of SLK, and provide targets for novel therapeutic approaches.

## Introduction

The Ste20-like serine/threonine protein kinase, SLK, is a member of the group five germinal center kinase family [1–3]. By analogy to other members of this family, SLK is, at least in part, a mitogen-activated protein kinase kinase kinase kinase (MAP4K). As reviewed recently [1], the physiological roles of SLK appear to be diverse, but remain incompletely understood. Global deletion of wild type (WT) SLK and replacement with an inefficiently expressed SLK mutant protein in mice resulted in severe developmental defects in the placenta and multiple tissues at embryonic day 12, leading to a lethal phenotype at day 14 [4], attesting to an important role of SLK in development. In cells, SLK can regulate apoptosis and cytokinesis [1,5,6]. In kidney, expression and activity of SLK were enhanced in ischemia-reperfusion injury in rats [7]. Overexpression of SLK was shown to induce apoptosis in cultured glomerular epithelial cells (GECs) and renal tubular cells [7], and to induce GEC/podocyte injury and proteinuria in vivo [8]. At a basal level of expression, SLK may play a role in cell cycle progression, based on the observation that SLK co-localizes with α-tubulin, particularly during metaphase re-assembly of the mitotic spindle [9]. Other effects of SLK in cells include dissolution of actin stress fibers and redistribution to the cell periphery, and loss of cell adhesion [1,10]. Several cytoskeletal proteins have been identified as substrates of SLK, including RhoA [11], ezrin [12,13], paxillin [14], and the p150^Glued^ dynactin subunit [15,16]. By modulating the cytoskeleton, SLK may control cell motility. The latter has been addressed mainly in fibroblasts, and it involves localization of SLK to the leading edge of cells, and is at least in part mediated by LIM only protein 4, as well as Src-family kinases [17,18]. Moreover, in keeping with these cytoskeletal actions, SLK-dependent phosphorylation of ezrin (a protein that interacts with filamentous actin and the plasma membrane), enabled localization of ezrin to the apical membrane of epithelial cells and regulated assembly of microvilli [12].

SLK is expressed in numerous tissues, including muscle, neuronal cells and kidney of the developing embryo [1,4,5,19]. In the adult kidney, SLK is expressed in tubular and glomerular epithelial cells [7]. As stated above, SLK expression and activity are increased during recovery from ischemia-reperfusion injury, which may recapitulate aspects of kidney development. In renal cells in culture, ischemia-reperfusion activated endogenous SLK resulting in signaling via p38 mitogen-activated protein kinase and enhanced apoptosis [20].

The regulation of SLK activity is complex, and may include mRNA stabilization, protein homodimerization, phosphorylation, and protein-protein interactions [21–25]. Under resting conditions, the activation segment of a protein kinase is typically unstructured [26]. Phosphorylation of the activation segment by upstream kinase(s) stabilizes the kinase in a catalytically competent conformation, which enhances catalytic activity and interaction with substrate(s), thereby allowing the downstream propagation of a signal [23,24,26,27]. Kinase activation can be facilitated by increasing the local concentration of the kinase relative to its substrate, e.g. by homodimerization [26,27]. Certain kinases can undergo autoactivation through activation segment self-phosphorylation [27,28]. In such kinases, the catalytic domain of one dimerization partner can phosphorylate the activation domain of the other partner, followed by reciprocal phosphorylation. The consensus phosphorylation sequence in the activation segment typically does not correspond to the substrate consensus sequence. Ultimately, there is activation of two kinase molecules, which then phosphorylate downstream targets [27,28].

The SLK protein consists of 1204–1235 amino acids, and contains a N-terminal catalytic domain (amino acids 34–292) and large C-terminal domain, which contains coiled-coils [5,29]. The C-terminal region of SLK can mediate homo- and heterodimerization [22,24]. Activation of SLK is also regulated by phosphorylation [5,6,20,23,27,30]. At least two potential phosphorylation sites, T183 and S189, are found in the activation segment of SLK [23,27]. In vitro, isolated SLK catalytic domains are able to form dimers, and induce phosphorylation of T183 and S189. These phosphorylations are believed to result in the formation of a hydrogen bond between K63 (the ATP binding site) and E79, thereby locking the SLK monomer into an active conformation, which can bind substrate [23,27]. In a previous study, we introduced serine and threonine mutations in the activation segment of SLK [23]. Compared with SLK WT, the T183A, S189A and T183A/S189A SLK mutants showed reduced kinase activity in vitro. Overexpression of WT SLK, but not the mutants, increased activation-specific phosphorylation of c-Jun N-terminal kinase and p38 kinase, as well as activating protein-1. We also showed that regulated dimerization of the SLK catalytic domain enhanced kinase activity, and this effect was abolished by mutating T183 and S189. The proapoptotic effect of overexpressed SLK was also blocked by mutating T183 and S189 [23].

In the present study, we further characterize and advance our understanding of the mechanisms of SLK activation. We demonstrate that dimerization of the catalytic domain enhanced autophosphorylation and exogenous kinase activity, i.e. ezrin phosphorylation. Mutation of the T183 and S189 phosphorylation sites reduced autophosphorylation and exogenous kinase activity. Mutations in the coiled-coil domain significantly reduced ezrin phosphorylation and tended to reduce autophosphorylation, implying that autophosphorylation can occur, at least in part, intramolecularly, but dimerization is required for exogenous kinase activity.

## Materials and methods

### Materials

Tissue culture media and Lipofectamine 2000 were from Invitrogen-Life Technologies (Burlington, ON) and Wisent (Saint-Jean-Baptiste, QC). DharmaFECT transfection reagent was from GE Healthcare (Mississauga, ON). Electrophoresis reagents were from Bio-Rad Laboratories (Mississauga, ON), and GE Healthcare. Rabbit anti-actin antibody (A2066) and calyculin A were purchased from Sigma-Aldrich Canada (Mississauga, ON). AP20187 was from Clontech Laboratories (Mountain View, CA). Erlotinib was purchased from Cayman Chemical (Ann Arbor, MI). Mouse anti-hemaglutinin antigen epitope tag (HA; sc-7392) and mouse anti-green fluorescent protein (GFP) antibodies were from Santa Cruz Biotechnology (sc-9996, Santa Cruz, CA). Rabbit anti-RRXpS/T (anti-phospho-protein kinase A substrate; 9624), which reacts with SLK phospho-S189 [23], rabbit anti-phospho-ezrin (T567)/radixin (T564)/moesin (T558; pERM; 3141), and rabbit anti-ezrin (3145s) antibodies were from Cell Signaling Technology (Danvers, MA). Rabbit anti-SLK and rabbit anti-SLK-phospho-T183 (pT183) antibodies were characterized previously [7,20,23,24]. Plasmids encoding full-length HA-SLK WT and full-length mutants, including T183A, S189A, T183A/S189A, I848G, L986G, and L986G/I989G, as well as full length myc-SLK K63R were described previously [6,20,22–24], as were plasmids HA-Fv-SLK 1–373 (WT), and mutants T183A/S189A, T193A, T183E, S189E, K63R, and E79A [23]. GFP-ezrin cDNA was obtained from Addgene (pHJ421, plasmid 20680) [31]. SLK siRNAs and the TriFECTa dicer-substrate RNAi transfection kit were obtained from Integrated DNA Technologies (Coralville, IA); siRNAs were directed against the following rat SLK target sequences: 5'-CAAGAGATAATTGAGAATAAAC, and 5'-AGCAACTTAAAGATCAGTATTTCAT. The scrambled siRNA (control; 5'-CGUUAAUCGCGUAUAAUACGCGUAT) does not recognize any sequences in human, mouse, or rat transcriptomes.

### Cell culture and transfection

Experiments were carried out in rat GECs, COS-1 monkey kidney cells and mouse C2C12 myoblasts [24]. Rat GECs were characterized previously [32], and were cultured in K1 medium (DMEM, Ham F-12, with 5% Nu-Serum and hormone mixture) [7,32]. COS-1 and C2C12 cells were cultured in DMEM with 10% fetal bovine serum [24]. Cells were seeded into tissue culture plates 24 h prior to transfection. Cells were transiently transfected 24 h after plating with plasmid DNAs, using Lipofectamine 2000, according to the manufacturer’s instructions. siRNA duplexes (20 nM) were transfected 24 h after plating of cells, using a TriFECTa kit and DharmaFECT reagent, according to the manufacturer’s instructions. Cells were studied 48 h after transfections.

For migration or wound healing assays, GECs were plated into chambers with inserts that allow establishment of ~500 μm gaps between cell monolayers (Ibidi USA Inc., Madison, WI). Migration was monitored upon removal of the inserts. Plates were photographed at serial intervals, and migration of cells into the gaps was quantified by measuring the widths of the gaps using National Institutes of Health ImageJ software. In preliminary experiments, it was demonstrated that the rates of migration were similar regardless of whether cells were pre-incubated in serum-poor culture medium for 12 h or in complete medium.

### Immunoprecipitation and immunoblotting

Cells were rinsed with PBS and lysed with buffer (“lysis buffer”), containing 1% Triton X-100, 125 mM NaCl, 10 mM Tris (pH 7.5), 1 mM EGTA, 2 mM Na_3_VO_4_, 5 mM Na_4_P3O_7_, 25 mM NaF, 20 μM leupeptin, 10 μM pepstatin, 50 μM bestatin, 15 μM E64, 0.8 μM aprotinin, 1 mM 4-(2-aminoethyl)benzenesulfonylfluoride. The lysates were then centrifuged at 13,000 g for 10 min. Immunoprecipitation of proteins in the supernatants was carried out by pre-clearance with agarose beads, followed by incubation with primary antibody or nonimmune IgG as control, and absorption of complexes using protein A-coupled agarose. After several washes, immune complexes were solubilized in Laemmli buffer and were subjected to SDS-PAGE [7,20,24].

For immunoblotting, after addition of Laemmli buffer to cell lysates, proteins were loaded onto gels with equal amounts of protein per lane. Proteins were separated by SDS-PAGE and were then electrophoretically transferred to a nitrocellulose or polyvinylidene fluoride membrane. Membranes were blocked with 5% BSA and incubated with primary antibody followed by horseradish peroxidase-conjugated secondary antibody. Membranes were developed with ECL. Density of specific bands was measured using ImageJ software. In preliminary studies, it was shown that there was a linear relationship between densitometric measurements and the amounts of protein loaded onto gels [7,20,23,24].

### Induction of passive Heymann nephritis (PHN)

Male Sprague-Dawley rats (150 g; Charles River, St. Constant, Quebec) were injected with 400 μl of sheep anti-rat Fx1A antiserum, as described previously [33]. This protocol resulted in the induction of proteinuria within 14 days of injection. Urine was collected from rats in metabolic cages for measurement of protein on days 13–14. Urine protein was quantified using the Bradford assay. Rats were euthanized on day 14, at which time kidneys were harvested for immunofluorescence staining. The study was carried out in accordance with guidelines established by the Canadian Council on Animal Care, and the animal protocol was approved by the McGill University Animal Care Committee (permit number 4475).

### Immunofluorescence microscopy

Kidney poles were snap-frozen in isopentane (-80°C). Cryostat sections (4 μm) were cut, air dried, fixed in 4% paraformaldehyde (15 min), and blocked with 10% normal goat serum (1 h, 22°C). Sections were incubated with a fluorophore-conjugated primary antibody (overnight, 4°C), or unconjugated primary antibody, followed by a fluorophore-conjugated goat anti-rabbit IgG (1 h, 22°C) [34]. Sections were examined with a Zeiss AxioObserver fluorescence microscope connected to an AxioCam digital camera. To allow comparisons of fluorescence intensities, all images were taken at the same exposure, with the length of exposure set to avoid camera saturation. Fluorescence intensity of glomeruli was quantified using the histogram function of Adobe Photoshop [34]. For all samples, a negative antibody control consisted of substitution of the primary antibody with nonimmune IgG.

### Statistics

Results are presented as mean ± standard error. One-way analysis of variance (ANOVA) was used to determine significant differences among groups. Where significant differences were found (ANOVA P<0.05), individual comparisons were then made between groups using the t statistic, and adjusting the critical value according to the Bonferroni method. The t-test was used to assess significant differences between two groups.

## Results

### Phosphorylation in the catalytic domain of SLK regulates kinase activity

**Fig 1:**
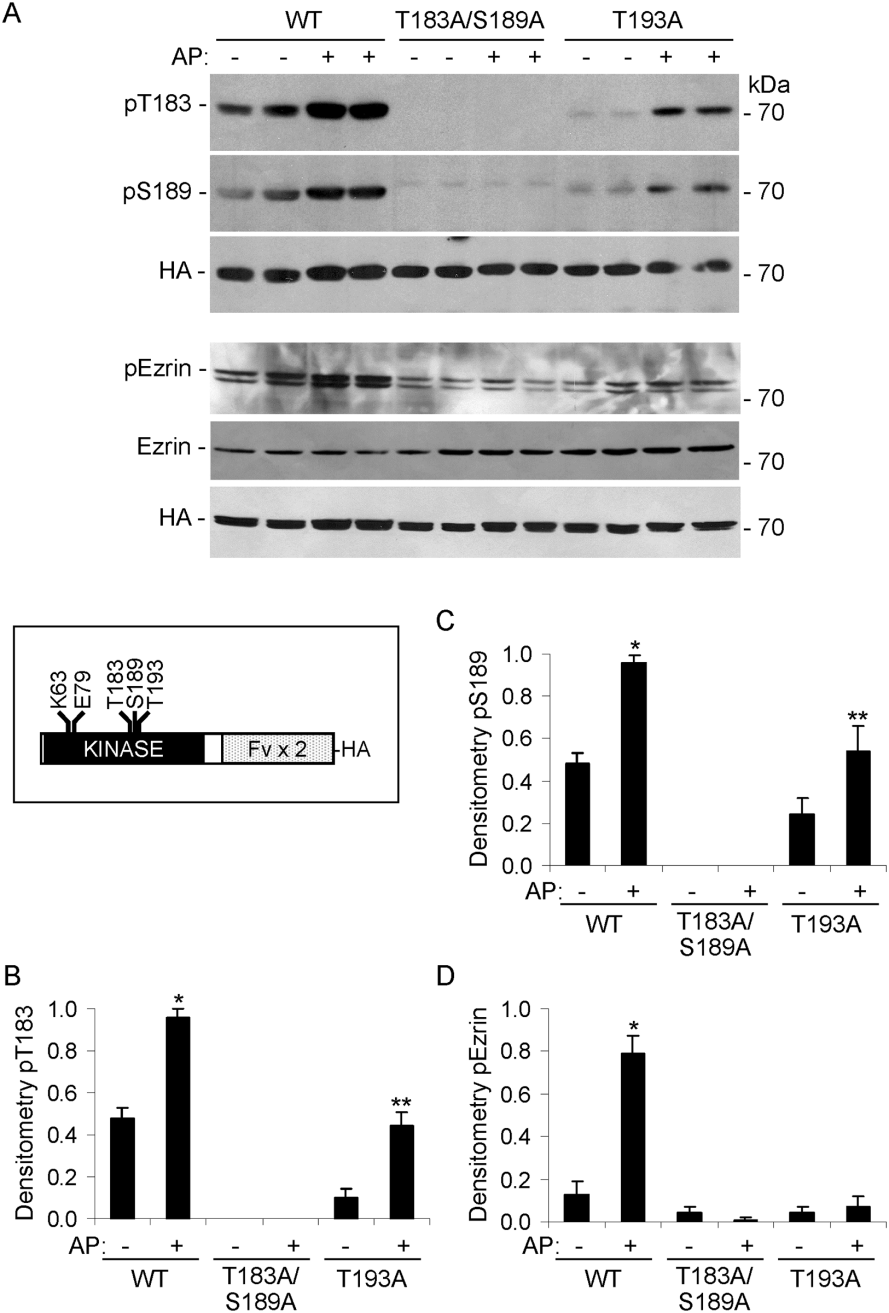
Phosphorylation in the catalytic domain of SLK regulates kinase activity. COS-1 cells were transiently transfected with HA-Fv-SLK 1–373 WT, T183A/S189A, or T193A. AP20187 (AP; 100 nM) was added as indicated at 24 h. Then, after 24 h, cell lysates were immunoblotted with anti-SLK pT183, anti-RRXpS/T (pS189), anti-HA, anti-pERM or anti-ezrin antibodies. T183 and S189 phosphorylation was increased after treatment of Fv-SLK 1–373 WT-expressing cells with AP20187. T183 and S189 phosphorylation was absent in the T183A/S189A mutant, and was reduced in the T193A mutant (the faint band in the S189 immunoblot is nonspecific). Phosphorylation of ezrin was abolished by T183A/S189A and T193A mutations (with and without AP20187). The lower band in the anti-pERM blot could represent an ezrin degradation product or moesin. A) Representative immunoblots. The inset illustrates the SLK catalytic domain showing the positions of ATP binding site at K63, E79, and the three phosphorylation sites. B-D) Densitometric quantification. B) *P<0.0001 AP vs untreated (WT) and WT vs T193A (AP), **P<0.0001 AP vs untreated (T193A). C) *P<0.0005 AP vs untreated (WT) and WT vs T193A (AP), **P<0.002 AP vs untreated (T193A). D) *P<0.0001 AP vs untreated (WT), WT vs T183A/S189A (AP), and WT vs T193A (AP). 4 experiments performed in duplicate.

In these experiments, we examined the role of dimerization and activation segment phosphorylation in the activation of SLK. We employed the Fv-SLK 1–373 fusion protein, which contains the kinase domain of SLK, fused with two copies of Fv (FK506 binding protein with a single amino acid substitution, F36V) (Fig 1). AP20187 (an analog of FK506) binds with subnanomolar affinity to Fv (while binding with 1000-fold lower affinity to the wild-type FK506 binding protein). By gel-filtration chromatography, we previously showed that AP20187 induces dimerization of Fv-SLK 1–373, and it enhances autophosphorylation and in vitro kinase activity [22,23]. We transfected COS-1 cells with HA-Fv-SLK 1–373 WT, T183A/S189A, or T193A, and then treated cells with AP20187. Autophosphorylation was monitored by immunoblotting with anti-SLK pT183 and anti-RRXpS/T (pS189) antibodies. Although we previously employed myelin basic protein to evaluate the exogenous kinase activity of SLK in vitro [23], since this earlier study, the full-length and catalytic domains of SLK were shown to induce phosphorylation of ezrin on T567 [12]. Therefore, in the present study, we evaluated the exogenous kinase activity of SLK using anti-pERM antibody, to identify the activating T567 phosphorylation of the physiological substrate, ezrin, in the context of intact cells. SLK T183 and S189 phosphorylation was increased after inducing dimerization of Fv-SLK 1–373 WT with AP20187 (Fig 1). As expected, T183 and S189 phosphorylation was absent in cells transfected with the T183A/S189A mutant, and was partially inhibited by the T193A mutation. Phosphorylation of ezrin was induced in the presence of Fv-SLK 1–373 WT, and was abolished completely by T183A/S189A and T193A mutations (with and without AP20187) (Fig 1). There was no significant effect of SLK activation on ezrin expression (Fig 1A).

**Fig 2:**
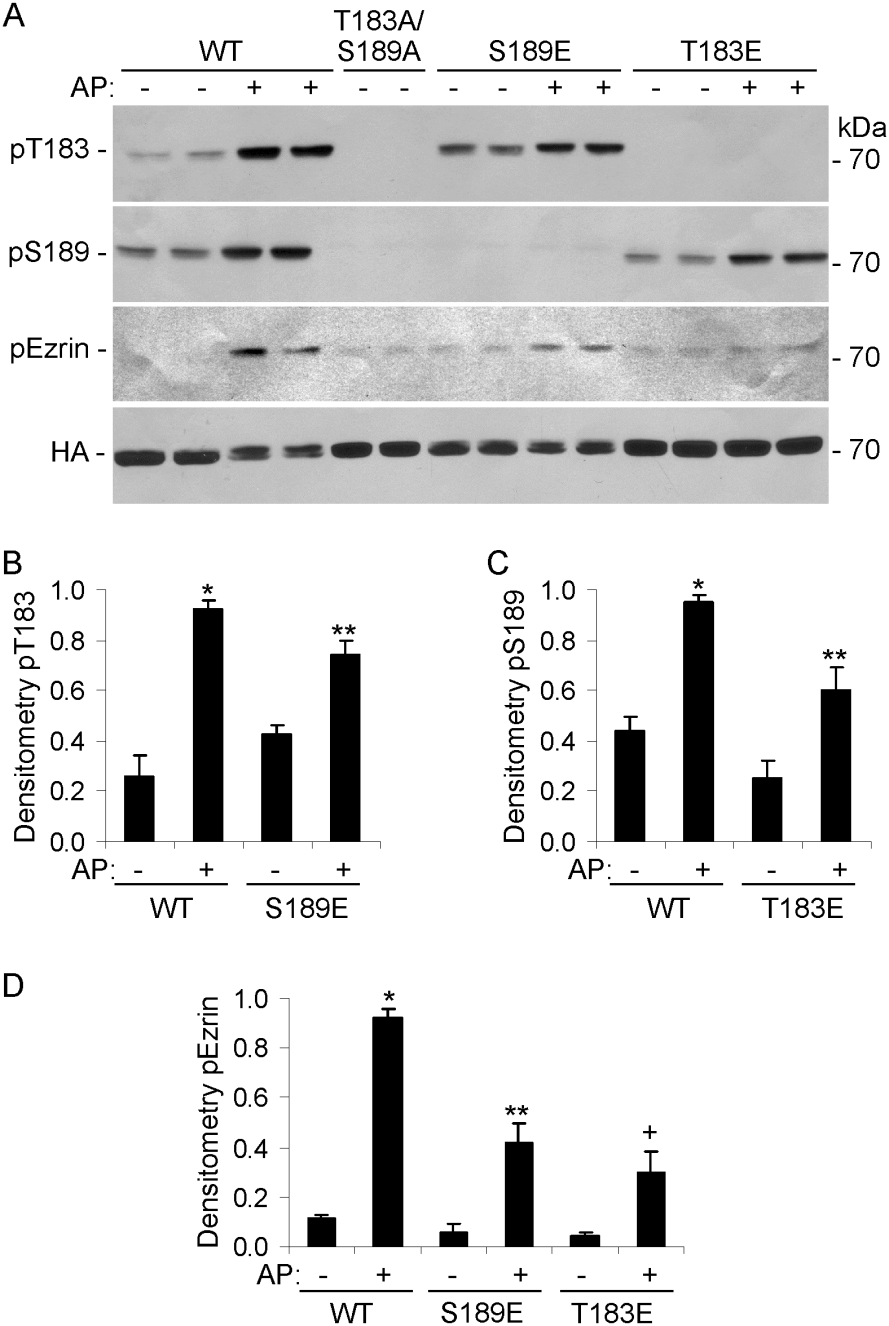
Role of T183 and S189 phosphorylation sites in the catalytic domain of SLK. COS-1 cells were transiently transfected with HA-Fv-SLK 1–373 WT, T183A/S189A, T183E, or S189E. AP20187 (AP; 100 nM) was added as indicated at 24 h. Then, after 24 h, cell lysates were immunoblotted with anti-SLK pT183, anti-RRXpS/T (pS189), anti-HA, or anti-pERM antibodies. T183 and S189 phosphorylation was increased after treatment of Fv-SLK 1–373 WT-expressing cells with AP20187. T183 and S189 phosphorylation was absent in the T183A/S189A mutant. T183 phosphorylation was reduced in the S189E mutant, and S189 phosphorylation was reduced in the T183E mutant. Phosphorylation of ezrin was reduced by both T183E and S189E mutations. A) Representative immunoblots. B-D) Densitometric quantification. B) *P<0.0001 AP vs untreated (WT) and P<0.015 WT vs S189E (AP), **P<0.0005 AP vs untreated (S189E). C) *P<0.0001 AP vs untreated (WT) and P<0.001 WT vs T183E (AP), **P<0.001 AP vs untreated (T183E). D) *P<0.0001 AP vs untreated (WT), WT vs S189E (AP), and WT vs T183E (AP), **P<0.0001 AP vs untreated (S189E), **P<0.0001 AP vs untreated (T183E). 5 experiments performed in duplicate.

In the next set of experiments, we examined the effects of individual mutations in T183 and S189 on phosphorylation of the corresponding site. COS-1 cells were transfected with HA-Fv-SLK 1–373 WT, T183A/S189A, T183E, or S189E. Cells were then treated with AP20187. In an earlier study, we had expected that substitution of alanine with glutamic acid would increase SLK catalytic activity, but we found that these mutations actually decreased in vitro kinase activity [23]. T183 and S189 phosphorylation was increased after treatment of Fv-SLK 1–373 WT-expressing cells with AP20187 (Fig 2). As expected, T183 and S189 phosphorylation was absent in the T183A/S189A mutant. T183 phosphorylation was partially inhibited by the S189E mutation, and S189 phosphorylation was partially inhibited by the T183E mutation (Fig 2). SLK-mediated phosphorylation of ezrin was partially reduced by both T183E and S189E mutations, compared with WT (Fig 2).

**Fig 3:**
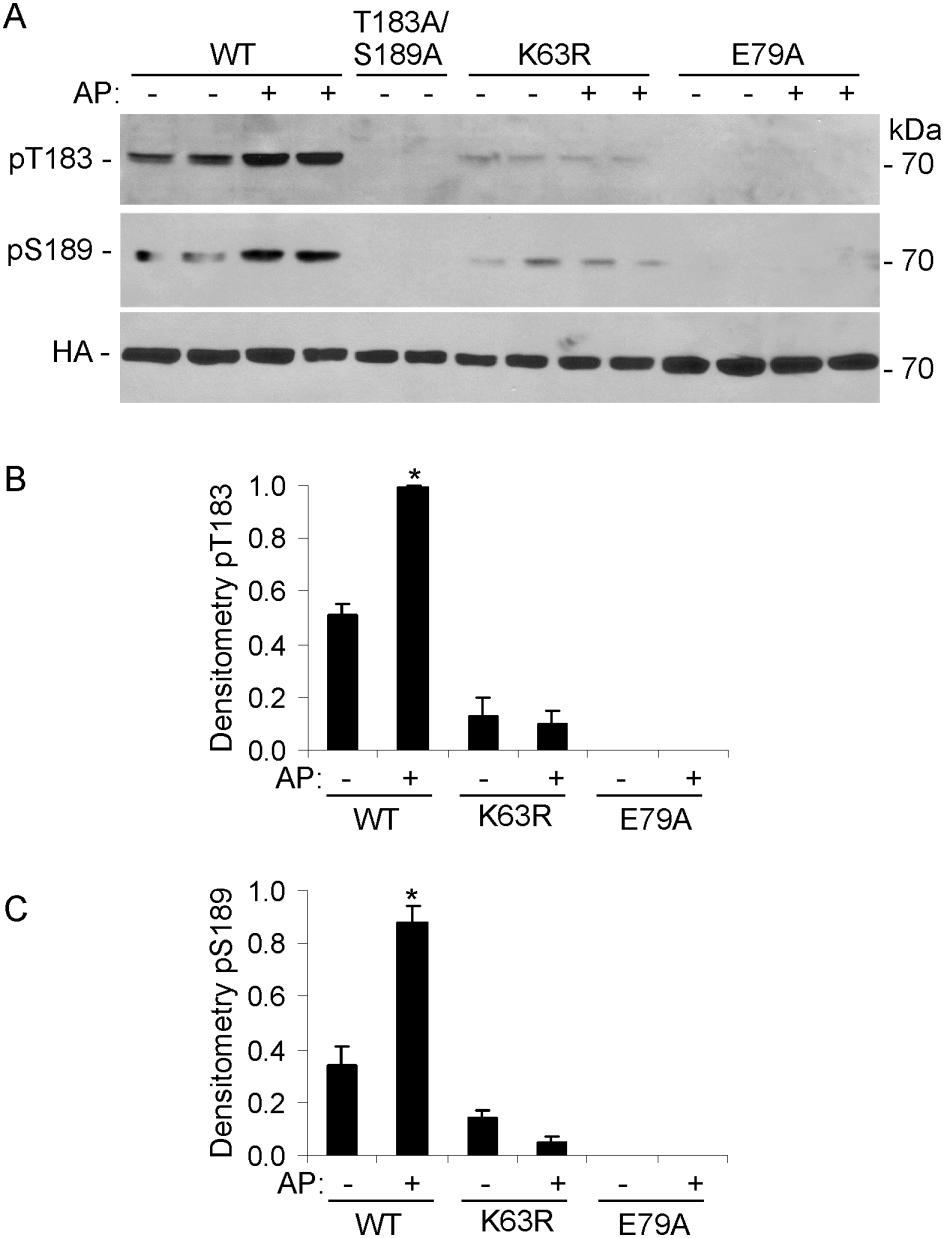
Role of K63 and E79 phosphorylation sites in the catalytic domain of SLK. COS-1 cells were transiently transfected with HA-Fv-SLK 1–373 WT, T183A/S189A, K63R, or E79A. AP20187 (AP; 100 nM) was added as indicated at 24 h. Then, after 24 h, cell lysates were immunoblotted with anti-SLK pT183, anti-RRXpS/T (pS189), or anti-HA antibodies. T183 and S189 phosphorylation was increased after treatment of Fv-SLK 1–373 WT-expressing cells with AP20187. T183 and S189 phosphorylation was absent in the K63R and E79A mutants. A) Representative immunoblots. B and C) Densitometric quantification. B and C) *P<0.0001 AP vs untreated (WT), WT vs K63R (AP), and WT vs E79A (AP). 4 experiments performed in duplicate.

To confirm that activation segment phosphorylation of SLK was actually dependent on its kinase activity, COS-1 cells were transfected with HA-Fv-SLK 1–373 WT, and the K63R kinase dead mutant. T183 and S189 phosphorylation was increased after treatment of Fv-SLK 1–373 WT-expressing cells with AP20187, but there was no significant phosphorylation of SLK K63R (Fig 3). This result also implies that T183 or S189 are not phosphorylated by another upstream protein kinase. SLK K63 is believed to form a bond with E79, which may lock SLK into an active conformation, suitable for substrate binding [27]. T183 and S189 phosphorylation was absent in the K63R and E79A mutants, implying a severe disruption of kinase activity (Fig 3).

### Phosphorylation sites involved in the regulation of SLK catalytic activity

**Fig 4:**
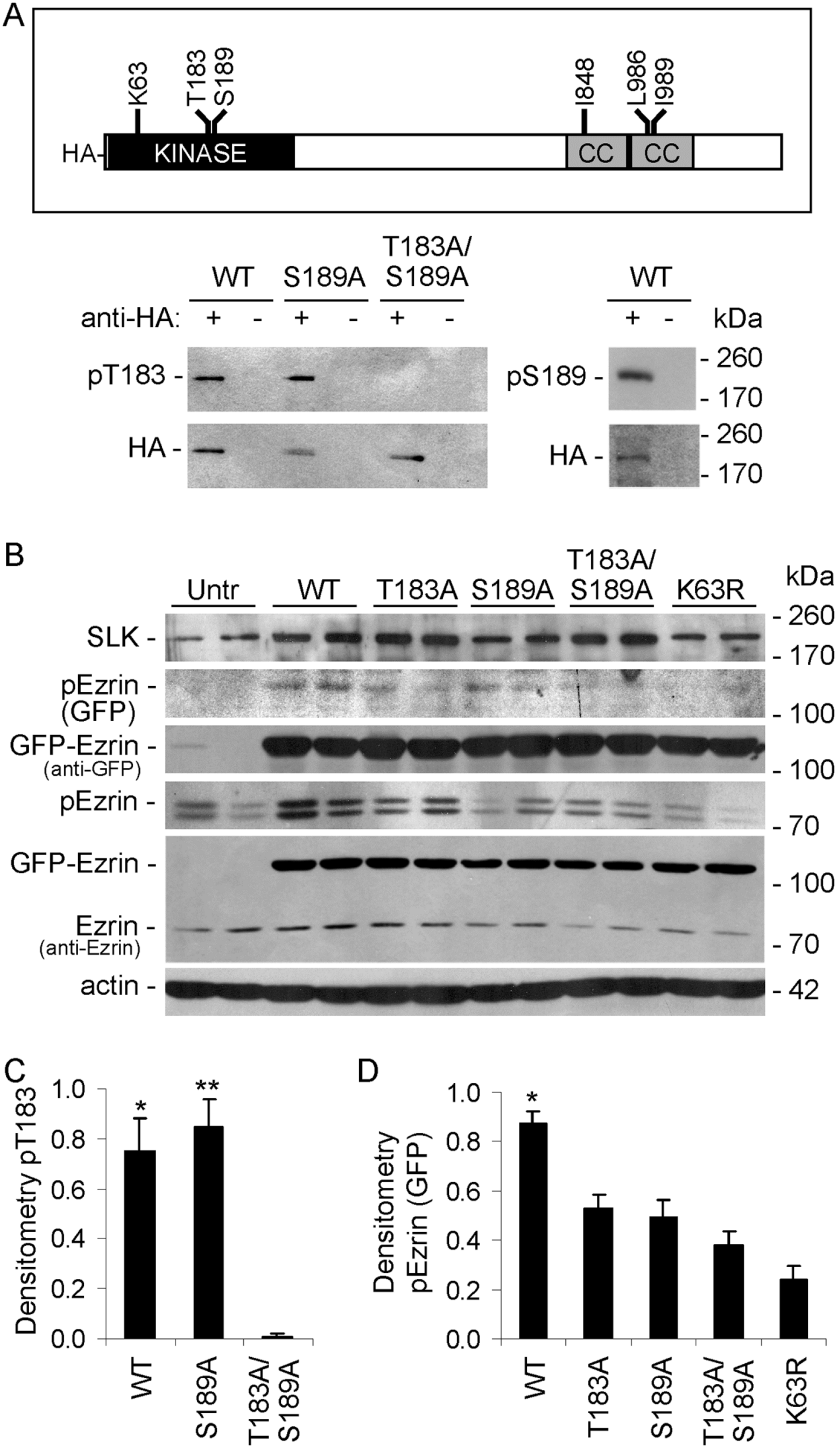
T183A, S189A, T183A/S189A and K63R mutations in the SLK activation domain reduce catalytic activity. A) Full-length HA-SLK (1204 amino acids). The kinase domain (amino acids 34–292), position of mutations, and coiled-coil (CC) domains are indicated. COS-1 cells were transiently transfected with WT or mutant full-length HA-SLK. After 48 h, lysates were immunoprecipitated with anti-HA antibody (+), or nonimmune IgG (control; -). Immune complexes were then immunoblotted with anti-pT183 or anti-HA antibodies. T183 phosphorylation was evident in the WT and S189 mutant. A) Representative immunoblots. C) Densitometric quantification. C) *P = 0.0001 WT vs T183A/S189A, **P<0.0001 S189A vs T183A/S189A. 5 experiments. B) COS-1 cells were transiently transfected with WT or mutants of full-length HA-SLK, as indicated, plus GFP-ezrin (Untr, untransfected control). After 48 h, lysates were immunoblotted with anti-SLK, anti-pERM, anti-GFP, anti-ezrin, or anti-actin antibodies. SLK mutants reduced ezrin phosphorylation. B) Representative immunoblots. D) Densitometric quantification. D) *P<0.0002 WT vs T183A, P<0.0001 WT vs S189A, P<0.0001 WT vs T183A/S189A, P<0.0001 WT vs K63R. 6 experiments performed in duplicate.

Having established a role for dimerization and activation segment phosphorylation in the activation of SLK using a regulated catalytic domain, we proceeded to investigate phosphorylation sites and kinase activity in the full-length protein (Fig 4A). COS-1 cells were transfected with WT, S189A or T183A/S189A full-length HA-SLK. Lysates were immunoprecipitated with anti-HA antibody and immune complexes were immunoblotted with anti-pT183. T183 phosphorylation was evident in both the WT and S189 mutant (Fig 4A and 4C). Also, immunoblotting of full-length HA-SLK WT immunoprecipitates with anti-pS189 antibody demonstrated S189 phosphorylation (Fig 4A).

Next, COS-1 cells were transfected with full-length SLK WT or T183A, S189A, T183A/S189A or K63R mutants. To verify the specificity of ezrin phosphorylation by SLK, cells were also transfected with GFP-ezrin. Transfection of full-length SLK WT stimulated phosphorylation of GFP-tagged and endogenous ezrin, while T183A, S189A, T183A/S189A mutations showed significantly lower ezrin phosphorylation (Fig 4B and 4D). As expected, ezrin phosphorylation was almost absent in the K63R mutant. GFP-ezrin was expressed ~5-fold above endogenous ezrin, and SLK activity did not alter expression of either protein (Fig 4B). Basal ezrin phosphorylation in untransfected COS-1 cells was reduced by 52±12% (P = 0.003) after transfection of SLK K63R. It should also be noted that transfection of HA-SLK increased the level of total SLK in COS-1 cells to 2.00±0.26-fold above endogenous (P<0.0001, 7 transfections performed in duplicate).

### Role of the coiled-coil domains in SLK activation

The SLK C-terminal domain is predicted to contain two coiled-coil regions, a N-terminal (amino acids 826–929) and a C-terminal coiled-coil (amino acids 942–1038) (Fig 4A) [24]. The SLK coiled-coils display typical heptad repeats with hydrophobic residues in the “a” and “d” positions [35]. To investigate the role of the SLK coiled-coil domains in mediating kinase activation, we introduced point mutations with the purpose of disrupting the coiled-coil structures. The I878G mutation, as well as the L986G and I989G mutations (all in the “a” or “d” positions) are predicted to disrupt the N-terminal and C-terminal coils, respectively [24,35]. Based on these predictions, we generated two single mutations in SLK, including I878G and L986G, as well as the double mutation, L986G/I989G. Earlier, we employed a protein fragment complementation assay to demonstrate that full-length SLK homodimerizes via the C-terminal domain [24]. In this assay, homodimerization was reduced by mutations including I848G, L986G and L986G/I989G (Fig 4A), conforming the in silico prediction [24].

**Fig 5:**
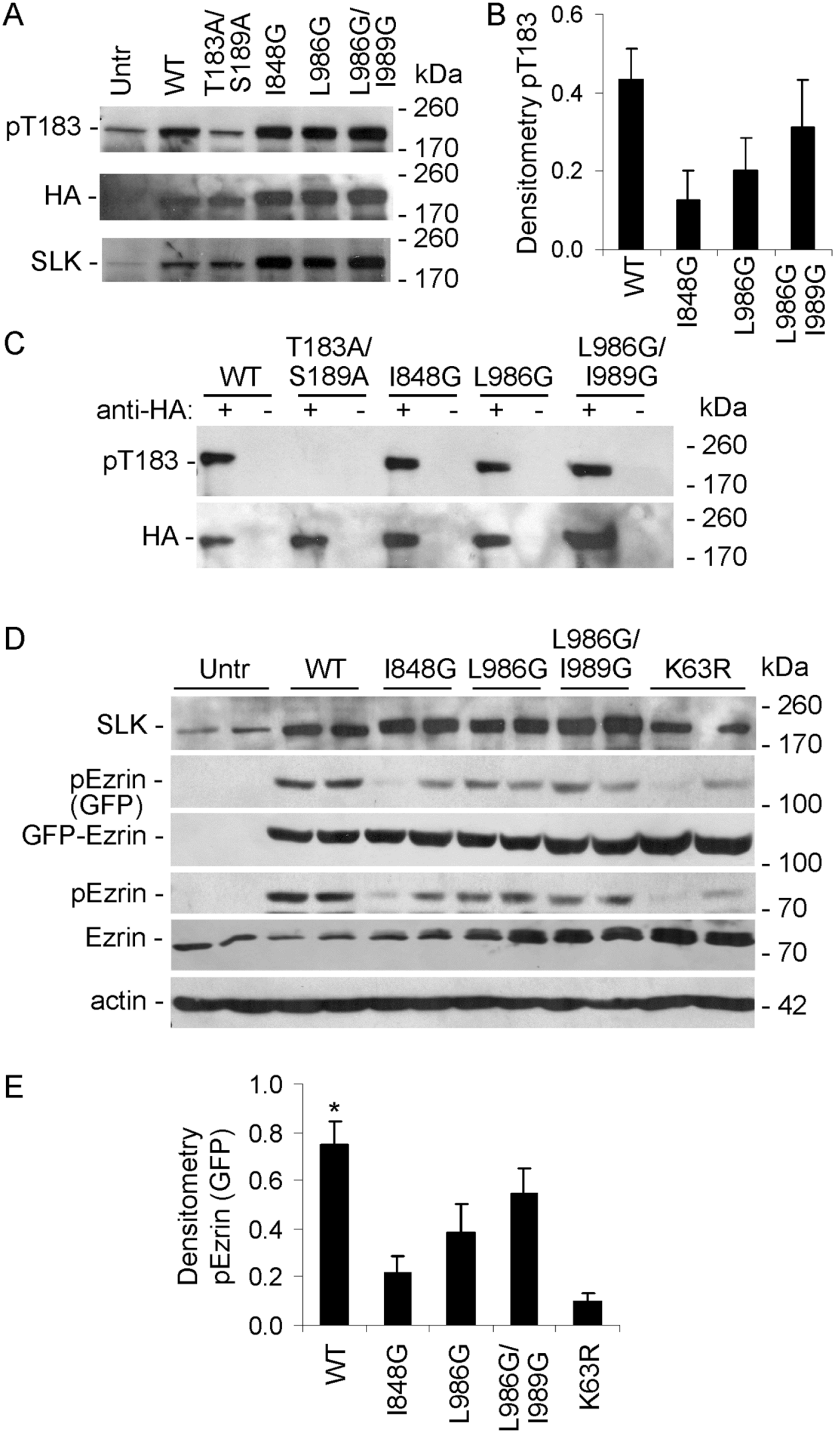
Coiled-coil domain mutations reduce SLK activity. A-C) COS-1 cells were transiently transfected with WT or mutants of full-length HA-SLK (Untr, untransfected control). A and B) After 48 h, lysates were immunoblotted with anti-pT183, anti-HA or anti-SLK antibodies. A) Representative immunoblots. B) Densitometric quantification. pT183 values were adjusted for HA-SLK expression, and background values (T183A/S189A mutant) were subtracted. T183 phosphorylation was evident in SLK WT and to a lesser extent in the coiled-coil domain mutants (I848G, L986G and L986G/I989G), although differences did not reach statistical significance. The minor T183 phosphorylation in Untr and the T183A/S189A mutant most likely reflects endogenous SLK. 5 experiments. C) COS-1 cells were transiently transfected as above. After 48 h, lysates were immunoprecipitated with anti-HA antibody (+), or nonimmune IgG (control; -). Immune complexes were then immunoblotted with anti-pT183 or anti-HA antibodies. T183 phosphorylation was absent in the T183A/S189A mutant, but was evident in the WT and coiled-coil domain mutants. Representative immunoblots. D) COS-1 cells were transiently transfected with WT or mutants of full-length HA-SLK, as indicated, plus GFP-ezrin (Untr, untransfected control). After 48 h, lysates were immunoblotted with anti-SLK, anti-pERM, anti-GFP, anti-ezrin, or anti-actin antibodies. SLK mutants reduced ezrin phosphorylation. D) Representative immunoblots. E) Densitometric quantification. E) *P<0.0005 WT vs I848G, P<0.03 WT vs L986G, P<0.0001 WT vs K63R. 6 experiments performed in duplicate.

In the present study, we examined the effects of coiled-coil domain mutations on SLK phosphorylation and kinase activity. COS-1 cells were transfected with WT or coiled-coil mutants of full-length HA-SLK. Immunoblotting with anti-pT183 antibody detected T183 phosphorylation in HA-SLK WT, as well as coiled-coil domain mutant transfections (I848G, L986G and L986G/I989G) (Fig 5A). The faint pT183 band in untransfected cells and in cells transfected with the T183A/S189A mutant (Fig 5A) most likely reflects phosphorylation of endogenous SLK (see below). However, the HA-SLK coiled-coil domain mutants showed greater expression levels, compared with WT, perhaps because of differences in plasmid construction. Thus, pT183 adjusted for HA-SLK expression tended to be lower in the coiled-coil domain mutants (I848G, L986G and L986G/I989G), although differences did not reach statistical significance, as there was considerable variability among experiments. To eliminate the confounding effect of endogenous SLK in the above experiments, we also monitored T183 phosphorylation after immunoprecipitation of HA-SLK. T183 phosphorylation was absent in the T183A/S189A mutant, but was evident in the WT and coiled-coil domain mutants (Fig 5C). By analogy to Fig 5A, a greater amount of coiled-coil domain mutant SLK was immunoprecipitated, compared with WT, implying that T183 phosphorylation was greater in SLK WT.

Finally, to monitor kinase activity, cells were transfected with WT or coiled-coil mutants of full-length HA-SLK, plus GFP-ezrin. Transfection of full-length SLK WT stimulated phosphorylation of GFP- and endogenous ezrin, while I848G, L986G and L986G/I989G mutations showed significantly lower ezrin phosphorylation (Fig 5D and 5E). As expected, ezrin phosphorylation was almost completely absent in the K63R mutant. Changes in GFP-ezrin phosphorylation were independent of expression, which did not change (Fig 5D). This pattern of ezrin phosphorylation parallels the pattern of SLK homodimerization, reported earlier [24]. Thus, the I848G mutation in the N-terminal SLK coil reduced dimerization and kinase activity most markedly, while the L986G and L986G/I989G mutations in the C-terminal coil were less disruptive.

### Kinase activity of endogenous SLK

**Fig 6:**
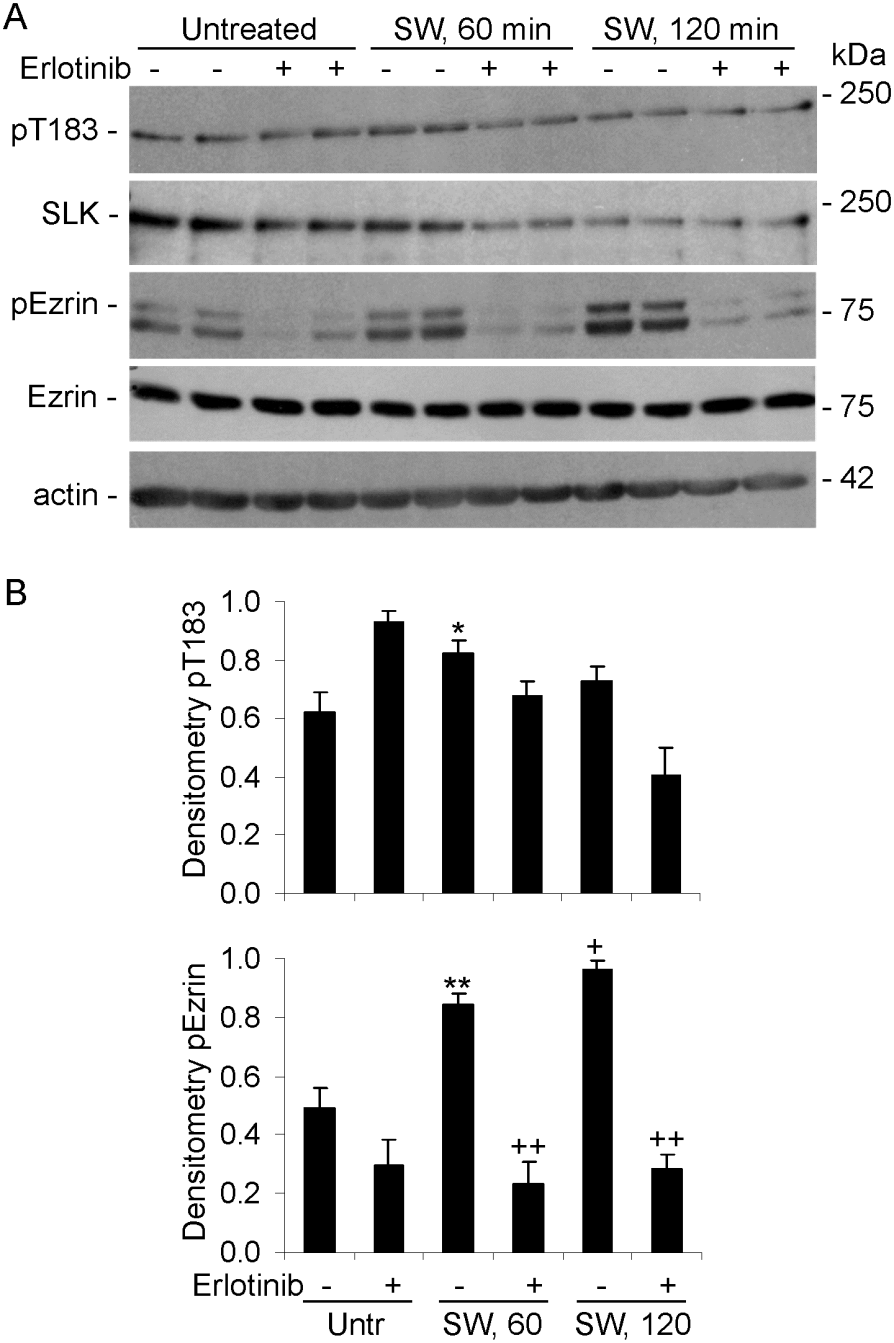
Scratch wounding enhances phosphorylation of endogenous SLK T183 and ezrin. C2C12 myoblasts were preincubated with (+) or without (-) erlotinib (10 μM) for 30 min, and were then untreated (Untr) or subjected to scratch wounding (SW). After 60 or 120 min, lysates were immunoblotted with anti-pT183, anti-SLK, anti-pERM, anti-ezrin, or anti-actin antibodies. A) Representative immunoblots. B) Densitometric quantification. B) *P<0.05, **P<0.005, ^+^P<0.0001 SW vs Untr (no erlotinib), ^++^P<0.0001 erlotinib vs no erlotinib, 3–10 experiments performed in duplicate.

The above experiments relied on expression of SLK WT and mutants in cells by transfection. To address the kinase activity of endogenous SLK, we initially employed C2C12 myoblasts, which express a ~3-fold higher level of endogenous SLK, compared with untransfected COS-1 cells [24]. It was previously reported that SLK activity may be stimulated by scratch wounding of fibroblasts [17,18]; thus, SLK activity was examined in untreated C2C12 myoblasts, and after subjecting the cells to scratch wounding. Immunoblotting of cell lysates with anti-pT183 antibody showed that there was basal phosphorylation of SLK at T183. Scratch wounding enhanced T183 phosphorylation at 60 min modestly, and pT183 declined by 120 min (Fig 6). Ezrin phosphorylation was evident in resting cells, and it increased markedly by scratch wounding at 60 min, and continued to increase at 120 min (Fig 6). There were no changes in ezrin expression (Fig 6A).

**Fig 7:**
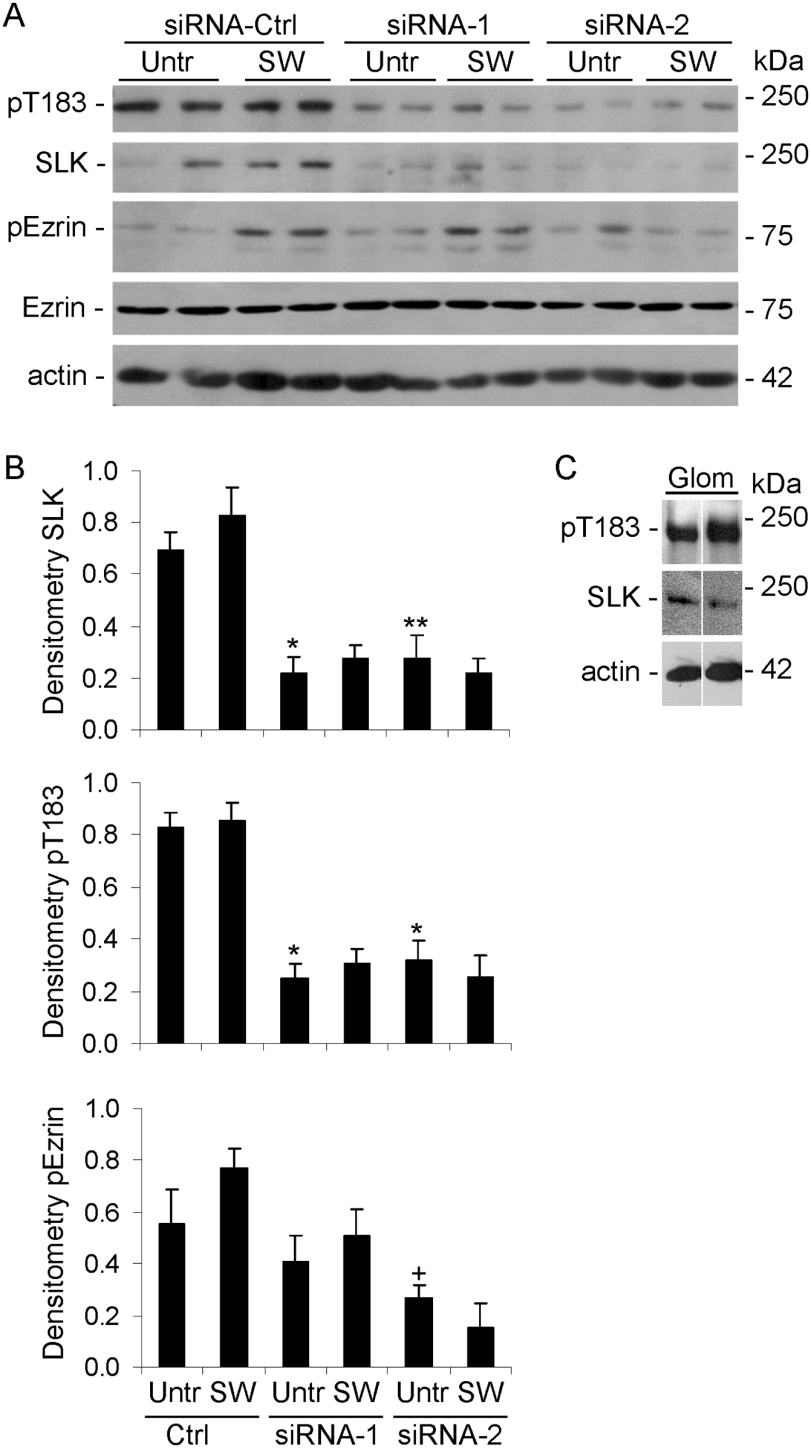
Activity/phosphorylation of endogenous SLK in GECs. GECs were transfected with two SLK-directed siRNAs or with scrambled control (Ctrl) siRNA. After 48 h, cells were untreated (Un) or subjected to scratch wounding (SW). After 1 h, lysates were immunoblotted with anti-pT183, anti-SLK, anti-pERM, anti-ezrin, or anti-actin antibodies. A) Representative immunoblots. B) Densitometric quantification. *P<0.0001, **P<0.0002, ^+^P<0.025 SLK siRNA vs Ctrl siRNA, 4 experiments performed in duplicate. C) SLK is constitutively phosphorylated in glomeruli. Glomeruli were isolated from normal rat kidneys, and were immunoblotted as indicated. Representative immunoblot showing glomeruli from 2 rats.

We have demonstrated important functional roles for SLK in kidney GECs in culture and in vivo [7,8]. Next, we used cultured GECs, a physiologically-relevant cell line, to examine phosphorylation of endogenous SLK and ezrin. Expression of SLK in GECs is significantly lower, compared with C2C12 cells. First, under basal conditions, SLK T183 was phosphorylated in GECs, and there was also detectable phosphorylation of ezrin (Fig 7A and 7B). By analogy, constitutive SLK T183 phosphorylation was evident in isolated rat glomeruli (Fig 7C). Glomeruli contain endothelial and mesangial cells in addition to GECs/podocytes, but SLK in the glomerulus is expressed prominently in the GECs [7]. Therefore, these results imply that basal phosphorylation of SLK in GECs in culture reflects the situation in vivo. Second, most studies that have addressed the role of SLK in cell migration have been performed in fibroblasts. GECs are not migratory cells when compared with fibroblasts, but it has been proposed that in vivo, GECs may nonetheless show some local motility within the glomerulus, and may display hypermotility after injury [36]. Given the link of cell motility with wound healing, we examined the effects of scratch wounding on changes in phosphorylation in cultured GECs. Cells were transfected with two SLK-directed siRNAs or with a scrambled control siRNA, and were then subjected to scratch wounding, or were untreated. Scratch wounding did not alter SLK pT183 significantly, although it tended to increase pERM by ~20% (Fig 7A and 7B). SLK-directed siRNAs reduced SLK expression and SLK pT183 by 67–68% (4 experiments performed in duplicate), and ezrin phosphorylation by 40–60% both in the presence and absence of scratch wounding (Fig 7A and 7B). This result implies that a significant portion of ezrin phosphorylation in GECs is dependent on SLK activity, but we cannot exclude the possibility that a basal component of ezrin phosphorylation is SLK-independent. We were not able to achieve knockdown of SLK beyond 68%, perhaps because these cells are difficult to transfect efficiently. Knockdown of SLK did not affect ezrin expression (Fig 7A).

### SLK catalytic activity and the effect of erlotinib

**Fig 8:**
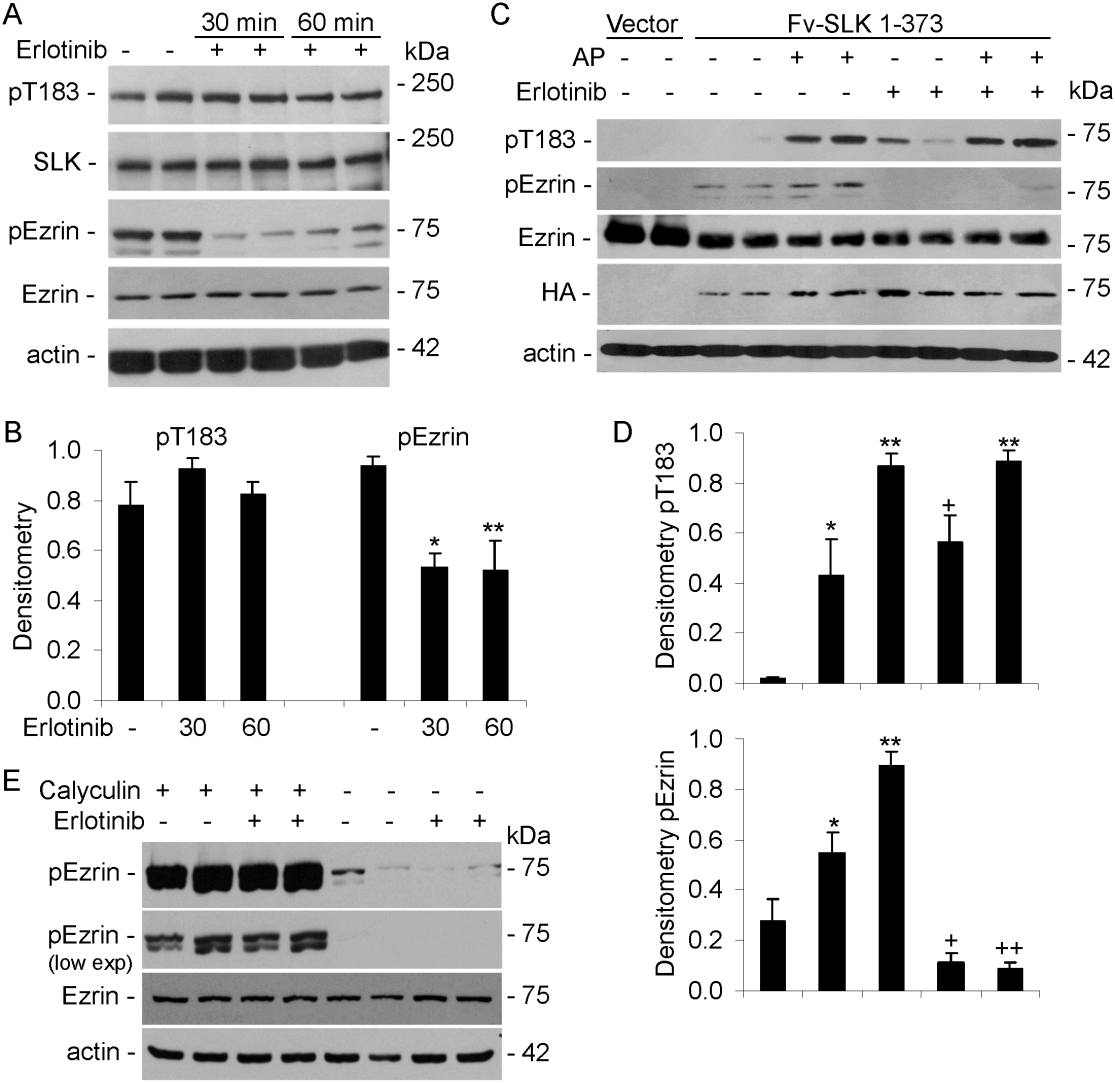
Erlotinib inhibits SLK-mediated ezrin phosphorylation, but not phosphorylation of SLK T183. A and B) GECs were incubated with (+) or without (-) erlotinib (10 μM) for 30 or 60 min. Lysates were immunoblotted with anti-pT183, anti-SLK, anti-pERM, anti-ezrin, or anti-actin antibodies. A) Representative immunoblots. B) Densitometric quantification. *P<0.015, **P = 0.005 erlotinib vs no treatment, 3 experiments performed in duplicate. C and D) COS-1 cells were transiently transfected with HA-Fv-SLK 1–373 WT or vector (V). AP20187 (AP; 100 nM) was added at 24 h, as indicated. Erlotinib (10 μM) was added at 48 h, for 60 min, as indicated. Lysates were immunoblotted with anti-SLK pT183, anti-HA, anti-pERM anti-ezrin, or anti-actin antibodies. C) Representative immunoblots. D) Densitometric quantification. pT183: *P<0.005, **P<0.0001, ^+^P<0.0005 vs vector; pEzrin: *P = 0.01, **P<0.0001 vs vector, ^+^P<0.0001 erlotinib vs untreated, ^++^P<0.0001 erlotinib+AP vs untreated+AP: 3 experiments performed in duplicate. E) Erlotinib does not directly inhibit calyculin A-induced phosphorylation of ezrin. GECs were preincubated with or without erlotinib (10 μM, 30 min), and were then incubated with calyculin A (50 nM, 30 min). Basal ezrin phosphorylation (i.e. without calyculin A) was, however, reduced by erlotinib, as in panel A. The anti-pERM immunoblot is representative of 3 experiments, and is presented at higher and lower exposures (low exp).

Erlotinib was originally developed as an inhibitor of the epidermal growth factor (EGF) receptor tyrosine kinase, but was later found to have a greater binding affinity for the kinase activity of SLK (and the related lymphocyte-oriented kinase), and to be an effective SLK inhibitor [12,37]. The drug appears to have a 5-10-fold lower affinity for Src-family kinases [37]. In C2C12 cells, erlotinib markedly inhibited ezrin phosphorylation induced by scratch wounding, but surprisingly, erlotinib did not produce any consistent effect on endogenous SLK pT183 (Fig 6). Actually, erlotinib tended to increase pT183 in the C2C12 cells not subjected to scratch wounding. Similarly, in GECs, erlotinib inhibited ezrin phosphorylation, but did not produce any consistent effect on endogenous SLK pT183 (Fig 8A and 8B). In both cell lines, phosphorylation of ezrin was not abolished by erlotinib entirely, suggesting that a basal portion of ezrin phosphorylation was SLK-independent. Also, erlotinib did not affect ezrin expression (Figs 6A and 8A). Therefore, the reduction in ezrin phosphorylation by erlotinib was distinct from the lack of erlotinib effect on SLK T183 phosphorylation. Moreover, the effect of erlotinib on ezrin phosphorylation was consistent with the effects of SLK-directed siRNAs (Fig 7).

To further delineate the specificity of erlotinib, COS-1 cells were transfected with Fv-SLK 1–373 WT. AP20187 was added to induce dimerization of the catalytic domain, in the presence or absence of erlotinib. In keeping with earlier experiments, there was some basal phosphorylation in SLK T183, and this was enhanced by AP20187 (Fig 8C and 8D). Neither basal nor stimulated T183 phosphorylation was blocked by erlotinib (Fig 8C and 8D). Expression of Fv-SLK 1–373 induced robust ezrin phosphorylation, and this was enhanced by AP20187 (Fig 8C and 8D). SLK-induced ezrin phosphorylation was abolished completely in the presence of erlotinib (Fig 8C and 8D). Thus, similarly to endogenous SLK in GECs and C2C12 cells, erlotinib did not affect Fv-SLK T183 phosphorylation; however, SLK-dependent ezrin phosphorylation was inhibited. Finally, to determine if erlotinib could directly inhibit T567 phosphorylation of ezrin, we induced robust ezrin phosphorylation using calyculin A (a protein phosphatase inhibitor) [12] in the presence or absence of erlotinib. Erlotinib showed no inhibitory effect on calyculin A-induced ezrin T567 phosphorylation (Fig 8E). This result indicates that erlotinib most likely does not interfere directly with ezrin phosphorylation.

### SLK mediates GEC motility

**Fig 9:**
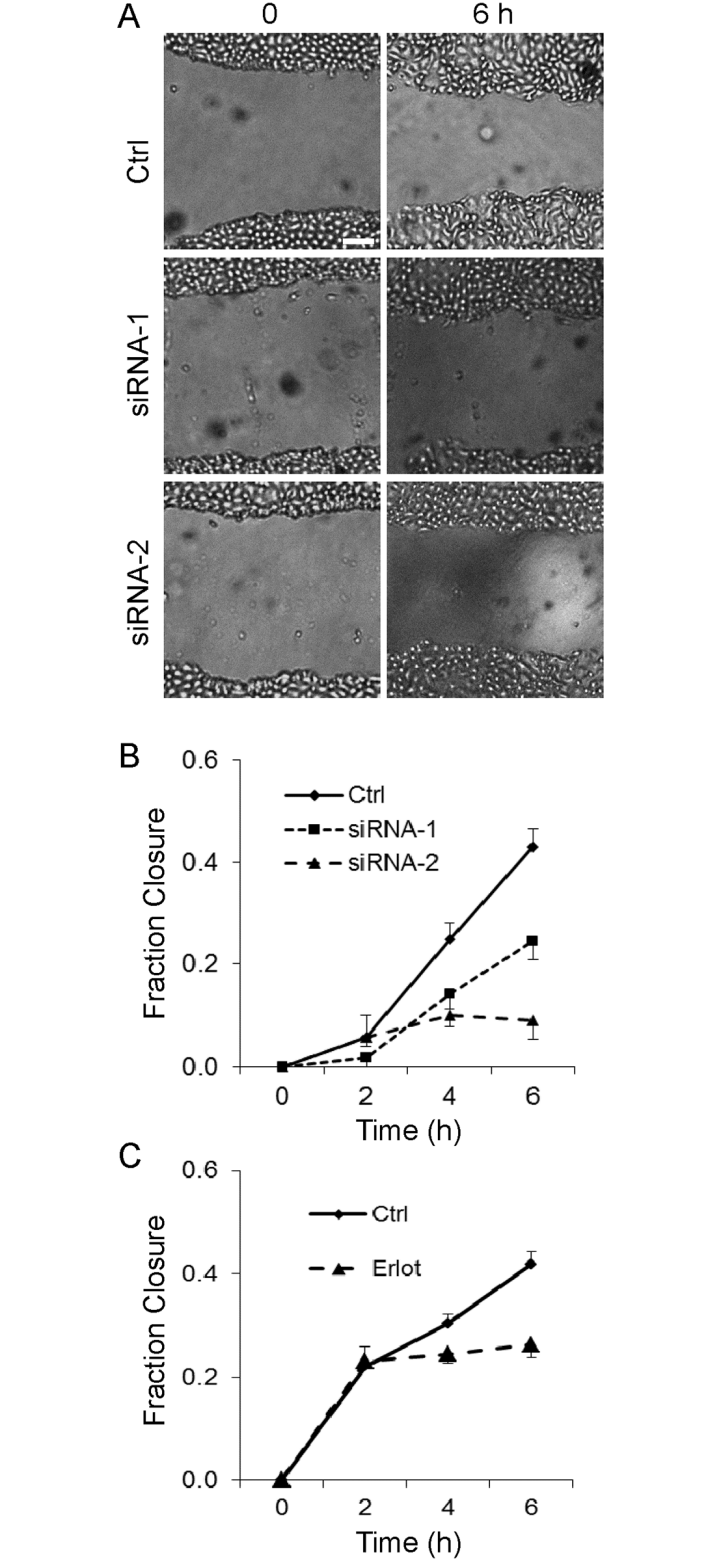
SLK mediates GEC motility. A, representative photomicrographs; B and C, quantification. GECs were plated into tissue culture chambers with inserts that allow establishment of ~500 μm gaps between cell monolayers. A and B) GECs were transfected with two SLK-directed siRNAs or with scrambled control (Ctrl) siRNA (as in Fig 7). Upon removal of the inserts at 48 h, migration was monitored for 6 h. Transfection of SLK siRNAs reduced the rate of GEC migration. P<0.005 siRNA-1 vs Ctrl at 6 h, P = 0.001 siRNA-2 vs Ctrl at 4 h, P<0.0001 siRNA-2 vs Ctrl at 6 h, 9–12 measurements. Bar = 100 μm. C) At 48 h erlotinib (10 μM) was added for 30 min to one group of cells. After removal of the inserts, migration was monitored for 6 h in the presence or absence of erlotinib. Erlotinib reduced the rate of migration. P<0.02, P<0.0001 erlotinib vs Ctrl at 4 and 6 h, respectively, 23 measurements.

As noted above, in vivo, GECs are phenotypically distinct from fibroblasts, but have been viewed as possessing a motile phenotype within the glomerulus, and perhaps becoming hypermotile after injury [36]. To link biochemical pathways of SLK activation with a functional output, we employed a wound healing assay. GECs were plated into chambers with inserts that allow establishment of ~500 μm gaps between cell monolayers. Upon removal of the insert, migration was monitored for up to 6 h. At 6 h, ~40% of the gap was covered by migrating GECs that had been transfected with scrambled siRNAs (Fig 9A and 9B). The gap was fully covered by these GECs within ~24 h (data not shown). Transfection of SLK siRNAs, under conditions that reduced SLK expression and ezrin phosphorylation (Fig 7A and 7B), attenuated the rate of GEC migration by 45–90% at 6 h, compared with the scrambled siRNA (Fig 9A and 9B). Furthermore, in the presence of erlotinib, which partially inhibited ezrin phosphorylation (Fig 8), migration of GECs was reduced by ~40%, compared with untreated control (Fig 9C; photomicrographs not shown).

### SLK is activated in PHN

**Fig 10:**
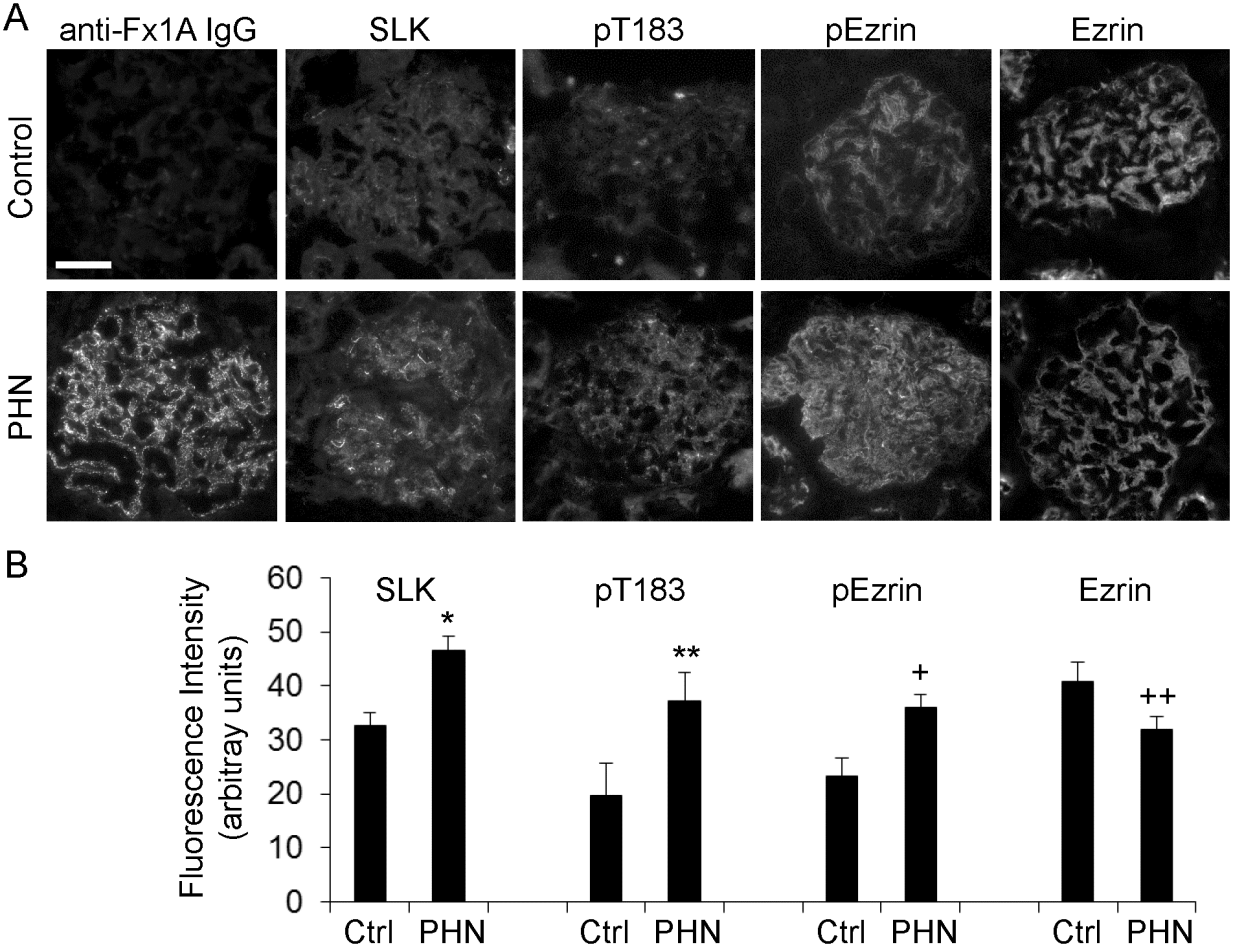
SLK is activated in PHN. Rats were untreated (Control, Ctrl), or injected with sheep anti-Fx1A antiserum to induce PHN. Kidneys were isolated on day 14, and kidney sections were incubated with primary antibodies, as indicated, followed by fluorophore-conjugated secondary antibody. A, representative photomicrographs; B quantification of fluorescence intensity. *P<0.0005, **P<0.04, ^+^P<0.01, ^++^P<0.05 PHN vs control (measurements were performed in 12–22 glomeruli in 4–6 rats per group). Bar = 50 μm. Incubation of kidney sections from control and PHN rats with nonimmune IgG (instead of primary antibody; negative control) resulted in an absence of immunofluorescent staining (results not shown).

To address SLK activation and phosphorylation in a pathophysiological context, we examined SLK activation in the PHN model of experimental membranous nephropathy, using quantitative immunofluorescence microscopy. In PHN, binding of anti-Fx1A antibody to GEC/podocyte plasma membranes leads to formation of immune deposits, complement activation and podocyte injury [33,38]. Fourteen days after induction of PHN, rats developed proteinuria (625±208 mg/24 h), compared with control (12±2 mg/24 h, P<0.05, 6–9 rats per group). Heterologous antibody (sheep anti-Fx1A IgG) was present in glomeruli of rats with PHN, but not control (Fig 10A). Glomerular SLK expression, and phosphorylation of SLK T183 and ezrin increased in rats with PHN, compared with control (Fig 10A and 10B), in keeping with glomerular SLK activation in PHN. Ezrin expression was actually decreased in PHN despite increased phosphorylation (Fig 10A and 10B). Along the glomerular capillary wall, ezrin is expressed primarily in podocytes and not endothelial cells, while moesin and radixin are expressed in endothelial cells, but not podocytes [39–41]. Consequently, the increase in pERM staining in PHN (a podocyte disease) most likely reflects an increase in podocyte phospho-ezrin.

## Discussion

The present study comprehensively addresses the activation and signaling of SLK in the context of intact cells, including a physiologically relevant cell line (GECs). Using phospho-specific antibodies and mutagenesis, the study demonstrates the important role of the SLK T183 and S189 phosphorylation sites in the activation segment, as well as critical amino acid residues in the coiled-coil regions. Controlled dimerization of the SLK catalytic domain enhanced activation segment autophosphorylation at T183 and S189 (Fig 1) [23,27]. Full-length ectopically- or endogenously-expressed SLK were constitutively phosphorylated at T183 and S189 (Figs 4–8). Using ezrin as a physiological SLK substrate (to address exogenous kinase activity), we demonstrated that dimerization of SLK 1–373 (Fig 1) or full-length SLK (Fig 5) is essential for effective ezrin T567 phosphorylation. Mutations in the coiled-coil region of full-length SLK, which impair dimerization [24], significantly reduced exogenous kinase activity and tended to reduce autophosphorylation of SLK at T183 (Fig 5).

Single mutations in T183, S189 or T193 partially reduced phosphorylation in the non-mutated activation segment sites, and showed greater reduction in ezrin phosphorylation (Figs 1 and 2). The effect of the T183A/S189A double mutation on blocking ezrin phosphorylation was almost complete and was greater than the effect of the two single mutations (Fig 1). In contrast to SLK 1–373, which is a constitutive monomer, we demonstrated previously that full-length SLK forms a high molecular mass complex in cells, which is a constitutive dimer (or oligomer) [22]. Unlike SLK 1–373, the S189A mutation in full-length SLK did not detectably reduce SLK T183 phosphorylation, compared with WT; however, S189A, T183A and T183A/S189A mutations in full-length SLK significantly reduced ezrin phosphorylation (Fig 4). These results suggest that a mutation of one phosphorylation site in the catalytic domain may lead to a modest impairment in phosphorylation of the other site, but the effect on exogenous kinase activity is more substantial. By analogy, it was reported that when assayed in vitro, the S189A mutation in the isolated catalytic domain did not affect T183 phosphorylation significantly, and that T183 is the primary phosphorylation site, while S189 is secondary [27]. The Drosophila SLK homolog, Slik, phosphorylates and activates moesin in developing epithelial tissues to promote epithelial integrity. Phosphorylation of at least two of three analogous conserved sites in the activation segment is required for efficient catalytic activity [42].

Previously, we employed a protein complementation assay to show that coiled-coil domain mutations reduced the ability of SLK to dimerize [24]. The present study provides additional key results, showing that SLK coiled-coil domain mutations reduced exogenous kinase activity, and tended to reduce T183 autophosphorylation (Fig 5). Interestingly, the I848G mutation, found in the N-terminal coiled-coil, had the most pronounced effect on reducing ezrin phosphorylation, while L986G and L986G/I989G were less effective (Fig 5). This result is in keeping with the protein complementation assay, which showed that I848G disrupted SLK dimerization more severely than L986G and L986G/I989G [24]. Based on these results, it can first be concluded that T183 and S189 phosphorylations are essential to kinase activity, but they may not reflect exogenous kinase activity precisely, since the sites can be at least partially phosphorylated, yet exogenous kinase activity is absent (Figs 4 and 5). In some previous studies, SLK autophosphorylation was used as an index of kinase activity, whereas the present study indicates that this may not be entirely accurate. Second, dimerization of SLK is essential for exogenous kinase activity, since coiled-coil mutations abolish exogenous kinase activity towards ezrin (Fig 5). Third, the coiled-coiled domains are likely not involved in the interaction of SLK with substrate, since ezrin was effectively phosphorylated by SLK 1–373 (Fig 1). This result is in keeping with an earlier study of ezrin phosphorylation [12], and with the mechanism of moesin phosphorylation by Drosophila Slik [42]. Actually, phosphorylation of T183 and/or S189 may be important for substrate interaction. The coiled-coil domain also appears to be involved in the recruitment of SLK, as well as Slik, to an apical localization in cells [12,42], and in mediating the interaction of SLK and Slik with other proteins [12,24,42].

The protein kinase inhibitor, erlotinib, was employed to further characterize the catalytic activity of SLK. Interestingly, erlotinib did not reduce T183 phosphorylation appreciably in full-length SLK, or in monomeric and dimeric SLK 1–373 (Figs 6 and 8). However, erlotinib markedly reduced phosphorylation of ezrin, in keeping with a previous study [12]. Although it is possible that the effect of erlotinib on ezrin phosphorylation was independent of SLK, we believe this is unlikely, since knockdown of SLK reduced ezrin phosphorylation (Fig 7), and phospho-ezrin increased in parallel with phosphorylation of T183 in SLK 1–373 (Fig 8), thereby linking SLK activity with ezrin phosphorylation. Furthermore, erlotinib did not block calyculin A-induced ezrin phosphorylation (Fig 8). Detailed studies on the inhibitory effect of erlotinib on the EGF receptor tyrosine kinase have provided insights into kinase inhibition [43]. The EGF receptor has at least 6 tyrosine autophosphorylation sites, and after stimulation of cells with EGF, there is a distinct temporal pattern of tyrosine autophosphorylation. The range of drug sensitivity for erlotinib varied substantially for each individual tyrosine residue autophosphorylation, and for phosphorylation of downstream signaling proteins. Erlotinib showed relatively limited inhibition of two EGF receptor phosphorylation sites [43]. This type of kinetic inhibition behavior suggests that erlotinib may be a noncompetitive inhibitor of ATP in the autophosphorylation of these EGF receptor tyrosines, and by analogy of SLK T183. Moreover, since erlotinib mainly blocked SLK substrate phosphorylation, but not T183 phosphorylation, the result suggests that erlotinib may block an interaction of the SLK catalytic domain with ezrin.

Based on crystallography studies, phosphorylation in the kinase activation segment, such as pT183 in SLK, is believed to stabilize the kinase in a conformation suitable for substrate binding [23,24,26,27]. In kinases that autoactivate, transient activation of the catalytic domain of one monomer can phosphorylate the activation domain of the partner monomer. In turn, activation of the partner leads to the phosphorylation of the original monomer in its activation segment. The result is the activation of two kinases, which can then phosphorylate downstream targets [27,28]. Kinases that may undergo such activation in addition to SLK include checkpoint kinase 2, death-associated protein kinase 3, and oxidative stress-responsive-1 [23,28,44]. In vitro, catalytic domain monomers show low-affinity interactions, and SLK and death-associated protein kinase 3 formed dimers in solution to a minor extent [23,27,44]. In intact cells, dimerization of the SLK catalytic domain appears to be constitutive, and is dependent on the C-terminal coiled-coil regions, as our protein complementation assay indicated that catalytic domains do not dimerize independently [20,24]. While dimeric SLK 1–373 showed phosphorylation at T183 and S189, the SLK 1–373 monomers also showed a minor degree of phosphorylation (Fig 1). Thus, SLK activation segment autophosphorylation could represent transient dimeric interactions of the catalytic domains, or alternatively our results point to the possibility of an intramolecular autophosphorylation mechanism. It has been suggested that S189 phosphorylation is relevant for monomeric kinase activity [27].

Phosphoproteomic analyses have identified S189 and T183 phosphorylation in endogenous SLK in multiple tissues/cells [45–50]. Other serine/threonine phosphorylation sites within and outside of the catalytic domain were also noted. In most cases, phosphorylation of T183 and S189 appears to be constitutive, and the extent to which further enhancement of phosphorylation at these sites leads to an increase in SLK activity is unclear. A study in HeLa cells showed ~2-fold variation in T183 and S189 phosphorylation during the cell cycle [45], in keeping with variations in SLK activity, reported earlier [30]. A 2-4-fold increase in S189 phosphorylation was induced by the stromal cell-derived factor 1/G protein-coupled receptor chemokine receptor 4-mediated pathway in breast cancer cells, which were used as a model of metastasis [49]. In the present study, SLK T183 phosphorylation was present in various resting cells and in normal glomeruli. In C2C12 cells, scratch wounding enhanced SLK T183 phosphorylation modestly, while ezrin phosphorylation was enhanced more markedly. Since SLK was previously shown to localize to the leading edge of fibroblasts and to mediate fibroblast migration (wound healing) after scratch wounding [17,18], these results support the view that enhanced phosphorylation regulates SLK activity and cell migration in this context. The so-called motile phenotype of GECs in culture is considered to be the in vitro analog of podocyte foot process effacement. In PHN, proteinuria and podocyte injury (which features foot process effacement) [33,38] were associated with increased SLK expression and activity (Fig 10). Together with the wound healing assay (Fig 9), these results indicate that SLK may play a role in the podocyte’s response to injury in glomerulonephritis [51] (also see below).

By analogy to other epithelia, in the kidney, SLK and ezrin phosphorylation may be important in maintaining the structure of microvilli on renal tubular epithelial cells [12]. GECs/podocytes in vivo show a more complex structure, compared with tubular epithelium. Ezrin is linked with the actin cytoskeleton, and is also linked with podocalyxin via Na^+^/H^+^-exchanger regulatory factor 2 (NHERF2). The ezrin-NHERF2-podocalyxin complex, situated on the apical side of the podocyte foot process, appears to be important in maintaining polarity and architecture [40,41,52], and could be a target of SLK, which was also reported to localize in the apical domain of epithelia [12]. Previously, SLK was shown to phosphorylate RhoA S188, which resulted in the inhibition of RhoA-mediated arterial contraction induced by angiotensin II type 2 receptor activation RhoA [11]. Both activation or inhibition of RhoA in podocytes may cause proteinuria [36,51,53]. How interactions of SLK with ezrin-NHERF2-podocalyxin or RhoA may be involved in the regulation of GEC cytoskeletal and foot process structure will require further study. Our preliminary studies show that deletion of SLK in GECs/podocytes in vivo leads to GEC injury and proteinuria, confirming a functionally important role for SLK in maintaining normal glomerular physiology [54]. The characterization of the key SLK phosphorylation sites and regulation of catalytic activity may also provide novel targets for design of drugs [27,55], which may be useful in the treatment of acute kidney injury, glomerulonephritis, wound healing, cancer, and other diseases.
